# Asthma: Gln27Glu and Arg16Gly polymorphisms of the beta2-adrenergic receptor gene as risk factors

**DOI:** 10.1186/1710-1492-10-8

**Published:** 2014-02-05

**Authors:** Ana Carolina Zimiani de Paiva, Fernando Augusto de Lima Marson, José Dirceu Ribeiro, Carmen Sílvia Bertuzzo

**Affiliations:** 1 Department of Medical Genetics, Faculty of Medical Sciences, State University of Campinas (Unicamp), Campinas, São Paulo zip code: 13081-970, Brazil; 2 Department of Pediatrics, Faculty of Medical Sciences, State University of Campinas (Unicamp), Tessália Vieira de Camargo, 126, Campinas, SP zip code: 13081-970, Brazil

**Keywords:** Asthma, *ADRB2* gene, Lung disease, Arg16Gly, Gln27Glu

## Abstract

**Background:**

Asthma is caused by both environmental and genetic factors. The *ADRB2* gene, which encodes the beta 2-adrenergic receptor, is one of the most extensively studied genes with respect to asthma prevalence and severity. The Arg16Gly (+46A > G) and Gln27Glu (+79C > G) polymorphisms in the *ADRB2* gene cause changes in the amino acids flanking the receptor ligand site, altering the response to bronchodilators and the risk of asthma through complex pathways. The *ADRB2* polymorphisms affect beta-adrenergic bronchodilator action and are a tool to identify at-risk populations.

**Objective:**

To determine the frequency of these two polymorphisms in allergic asthma patients and healthy subjects and to correlate these data with the occurrence and severity of asthma.

**Methods:**

Eighty-eight allergic asthma patients and 141 healthy subjects were included in this study. The *ADRB2* polymorphisms were analyzed using the amplification-refractory mutation system – polymerase chain reaction (ARMS-PCR) technique. The statistical analysis was performed with the SPSS 21.0 software using the Fisher’s Exact and χ^2^ tests.

**Results:**

The *ADRB2* polymorphisms were associated with asthma occurrence. The Arg16Arg, Gln27Gln and Gln27Glu genotypes were risk factors; the odds ratios were 6.782 (CI = 3.07 to 16.03), 2.120 (CI = 1.22 to 3.71) and 8.096 (CI = 3.90 to 17.77), respectively. For the Gly16Gly and Glu27Glu genotypes, the odds ratios were 0.312 (CI = 0.17 to 0.56) and 0.084 (CI = 0.04 to 0.17), respectively. The haplotype analysis showed that there were associations between the following groups: Arg16Arg-Gln27Gln (OR = 5.108, CI = 1.82 to 16.37), Gly16Gly-Glu27Glu (OR = 2.816, CI = 1.25 to 6.54), Arg16Gly-Gln27Glu (OR = 0.048, CI = 0.01 to 0.14) and Gly16Gly-Gln27Glu (OR = 0.1036, CI = 0.02 to 0.39). The polymorphism Gln27Glu was associated with asthma severity, as the Gln27Gln genotype was a risk factor for severe asthma (OR = 2.798, CI = 1.099 to 6.674) and the Gln27Glu genotype was a protective factor for mild (OR = 3.063, CI = 1.037 to 9.041) and severe (OR = 0.182, CI = 0.048 to 0.691) asthma.

**Conclusions:**

The Arg16Gly and Gln27Glu polymorphisms in the *ADRB2* gene are associated with asthma presence and severity.

## Background

Asthma is a chronic inflammatory disease of the airways defined by clinical, physiological and pathological characteristics. The main traits of allergic asthma in children are shortness of breath, wheezing, obstruction and inflammation of airways, and atopy [1]. Genetically, asthma is a complex disease in which multiple genes interact among themselves and with the environment [1].

Asthma affects approximately 300 million people worldwide (1 to 18% of the population in different countries) [2,3] and is associated with 250,000 deaths per year. In Brazil, 20% of the population is affected, with approximately 350,000 hospitalizations per year or 2.3% of the hospital admissions in the Public Health System [4]. Asthma-related mortality has been growing over the last 10 years but does not correlate with disease prevalence. Asthma causes 5 to 10% of the respiratory-related deaths, with a high number of deaths occurring at home [4].

There are several factors that influence the development of asthma, including genes that predispose an individual to atopy and airway hyperresponsiveness; obesity; sex; and environmental causes, such as allergens (house dust mites, animal fur, and fungi), viral infections, occupational sensitizers, tobacco smoke, air pollution and eating habits. Additionally, some immunological characteristics, such as immune system maturation and the number of exposures to infectious agents during the first years of life, are factors that affect the risk of developing asthma. Another characteristic linked to an increased risk of asthma is ethnicity, which reflects vast genetic differences as well as significant social and economic variations that affect exposure to allergens and access to health services [1,5-10].

Asthma severity is assessed by analyzing the frequency and intensity of symptoms and examining pulmonary function. Based on these criteria, asthma is classified as either intermittent or persistent asthma, the latter of which can be mild, moderate or severe [1].

The pathophysiological characteristic present in asthmatic patients is bronchial inflammation, which is the result of complex interactions between the inflammatory cells, cell-derived mediators, and airway cells [11].

An important factor studied in asthma-related research is the beta-2-adrenergic receptor, which is encoded by the *ADRB2* gene [12]. The *ADRB2* gene is a small gene on chromosome 5q31-q32 [13], a region genetically linked to asthma [14]. Nine coding polymorphisms were originally described in the *ADRB2* gene, including four that cause non-synonymous changes in the amino acid sequence (Gly16Arg, Gln27Glu, Val34Met and Thr164Ile).

The β2 receptors (β2-AR) are widely expressed in the respiratory tract, particularly in the airway smooth muscles [12,15-17]. They are members of a family of seven-transmembrane receptors [18] and are 413 amino acids long [19]. Once activated, the most clinically relevant effect of the β2-ARs in the pulmonary smooth muscle is relaxation, which may be caused by β2-AR agonists. Chronic exposure to these agonists leads to a significant reduction in the number of β2-ARs on the cell surface [16,17]. This down-regulation is reflected in vivo as a tolerance to the effects of the β2-AR agonists [20-24].

In airway smooth muscle cells, the β2-AR agonists activate adenylyl cyclase through membrane-coupled G-proteins; this activation increases the intracellular cAMP (cyclic adenosine monophosphate) concentration and relaxes the airway tonus [25]. The β2-AR agonists may also affect Ca^2+^ and K^+^ channels in smooth muscles and lead to relaxation independently of cAMP [26].

The two most frequent deleterious polymorphisms in the *ADRB2* gene are Arg16Gly (+46A > G; rs1042713) and Gln27Glu (+79C > G; rs1042714). The Arg16Gly and Gln27Glu polymorphisms are near the receptor’s ligand-binding site [27]. The frequency of Gly16 is greater than that of Arg16, which is considered the normal allele. The allelic frequency described for the Arg16 variant ranges from 67% to 72% in different populations [28,29].

In the Brazilian population, to the best of our knowledge, there are no studies on asthma and the frequency of the Arg16Gly and Gln27Glu polymorphisms that take into account asthma risk and clinical severity. Therefore, our study included asthma patients and healthy subjects, and the associations between both groups and each polymorphism were assessed during the same analysis. The clinical evaluation of asthma severity was associated with the Arg16Gly and Gln27Glu polymorphisms.

## Methods

### Patients and healthy controls

A cross-sectional prospective study including 88 asthmatic patients was conducted at the Pediatric Pulmonology Clinic at the University Hospital.

The mean age was 10.38 (±2.93) years with a range of 7 to 16 years. All patients enrolled had allergic asthma according to the GINA criteria [1]. The allergy classification was defined by co-occurrence with asthma, atopic dermatitis, a positive skin test in response to allergens (dust mites, fungi, or house dust components), increased IgE serum levels, greater than 4% eosinophils in the peripheral blood in the absence of parasites and clinical history. All patients were subjected to three parasitological stool examinations three months prior to the onset of the study and were treated with albendazole as necessary.

The control group was composed of 141 healthy subjects ranging in age from 18 to 25 years who donated blood at the Unicamp University Hospital. In our data, all controls were examined for allergic asthma and a family history of asthma. In the case of a family history of asthma, the subject was excluded from our control group.

The project was approved by the University Ethics Committee (#267/2005), and all patients and/or their guardians signed an informed consent.

### Arg16Gly and Gln27Glu polymorphism analyses

Genomic DNA was extracted from the venous blood samples using phenol-chloroform. The DNA concentration was determined using a GE NanoVue™ Spectrophotometer (GE Healthcare Biosciences, Pittsburgh, USA), and 50 ng/mL of each sample was used for the analysis.

Polymorphism analysis of the *ADRB2* gene was performed by the polymerase (PCR) allele specific (ARMS) reaction [30,31]. Four reactions were performed (ARMS1a, ARMS2a, ARMS1b and ARMS2b), each containing a common primer (5′-AGG CCC ATG ACC AGA TCA GCA CAG GCC AG-3′) and one allele-specific primer [ARMS1a (5′- ACG GCA GCG CCT TCT TGC TGG CAC CCA AAA-3′), ARMS2a (5′-ACG GCA GCG CCT TCT TGC TGG CAC CCA AAG-3′), ARMS1b (5′-GCC ATG CGC CGG ACC ACG ACG TCA CGC ATC-3′) and ARMS2b (5′-GCC ATG CGC CGG ACC ACG ACG TCA CGC AAG-3′)]. All four reactions were performed under the same conditions. Each 10 μL reaction contained 1 × 4 PCR buffer, 200 μM of dNTPs, 5.0 nM of MgCl_2_, 0.4 U of Taq polymerase, 0.2 pmol of each primer and 1.0 μL (approximately 50 ng) of genomic DNA.

The PCR amplification conditions consisted of 5 minutes at 94°C followed by 35 cycles of 94°C for 1 minute, 60°C (46A or G, 16Arg or Gly) or 67°C (70C or G, 27 Gln or Glu), and 72°C for 1 minute followed by 72°C for 10 minutes.

The amplicons were subjected to electrophoresis on a 12% acrylamide gel and stained with ethidium bromide.

### Statistical analysis

Statistical analysis was performed using the software SPSS (Statistical Package for the Social Sciences) version 21.0 (Armonk, NY: IBM Corp), Open Epi [32] and R version 2.12 (Comprehensive R Archive Network, 2011). The statistical power calculation for the sample was performed using the GPOWER 3.1 software [33] and demonstrated statistical power above 80% for the analysis conducted. An alpha level of 0.05 was used in all of the data analyses.

The Fisher’s Exact and chi-squared (χ^2^) tests were performed to determine the association between the polymorphisms analyzed and the presence and severity of asthma.

The Hardy-Weinberg equilibrium was calculated using the Online Encyclopedia for Genetic Epidemiology (OEGE) software (http://www.oege.org/software/hardy-weinberg.html).

To calculate the sample power, the GPower*3.1.6 program was used [33]. In the calculation, we considered the minor allele frequency (MAF) to establish the sample size. According to the NCBI (National Center for Biotechnology Information - http://www.ncbi.nlm.nih.gov/) database, the frequencies of the A and C alleles at the 46A > G and 79C > G polymorphisms were 0.471 and 0.238, respectively. With the frequency of 0.238, α = 0.05 and β = 0.80, the power calculation estimates the patient sample size should be 193 patients based on using a χ^2^ test for the comparisons to be performed. In our study, we included 229 patients and controls and with our population obtained a β-error of 0.846.

To evaluate the genetic interactions among the polymorphisms in our sample, we used the Multifactor Dimensionality Reduction (MDR) model, which is a nonparametric and genetic model-free data mining tool for nonlinear interaction identification among genetic and environmental attributes [34-36]. To adjust the results for multiple comparisons, we performed a MDR permutation test on our data using 100,000 permutations.

## Results

The allelic frequencies for the Arg16Gly polymorphism were 94 (53.4%) and 82 (46.6%) for the A and G alleles, respectively, in the asthma group and 77 (27.3%) and 205 (72.7%), respectively, in the healthy subjects. For the Gln27Glu polymorphism, the allelic frequencies for the C and G alleles were 118 (67.0%) and 48 (33.0%), respectively, in the asthma group and 93 (33.0%) and 189 (67%), respectively, in the healthy subjects.

The polymorphisms are in Hardy-Weinberg equilibrium except for the Gln27Glu polymorphism, which is not in equilibrium in the healthy subject population. The complete genotype data and the Hardy-Weinberg equilibriums are shown in Table 1.

**Table 1:** **Association of *ADRB2 *** **polymorphisms [Arg16Gly (c.46A > G) and Gln27Glu (c.79C > G)] with asthma risk**

**Polymorphism**	**Genotype**	**Patient**	**Control**	**OR**	**95% CI**
**Arg16Gly (c.46A > G) **^**1,2**^	Homozygous Arg16	28	9	**6.782**	**3.07-16.03**
	Heterozygous	38	59	1.056	0.61-1.81
	Homozygous Gly16	22	73	**0.312**	**0.17-0.56**
	**Total**	**88**	**141**		
**Gln27Glu(c.79C > G) **^**3,4**^	Homozygous Gln27	41	41	**2.120**	**1.22-3.71**
	Heterozygous	36	11	**8.096**	**3.90-17.77**
	Homozygous Glu27	11	89	**0.084**	**0.04-0.17**
	**Total**	**88**	**141**		

*ADR2* = alpha2-adrenergic receptor; Arg = arginine; Gly = Glycine; Gln = Glutamine; Glu = Glutamic acid; A = Adenine; G = Guanine; T = Thymine; OR = Odds Ratio; CI = Confidence Interval.

Hardy-Weinberg equilibrium was calculated using the OEGE tool (http://www.oege.org/software/hardy-weinberg.html), being: (1 - patient) χ^2^ = 1.54, p-value > 0.05; (2 - control) χ^2^ = 0.48, p-value > 0.05; (3 - patient) χ^2^ = 0.41, p-value > 0.05; (4 - control) χ^2^ = 95.62, p-value < 0.001.

The OR was calculated using the Open-Epi tool (http://www.openepi.com). The OR and 95% CI values were obtained using the Mild-P test. For all data analyzed, α = 0.05 was used. The significant p-values are in bold.

In our data, the *ADRB2* polymorphisms were associated with the occurrence of asthma. For the Arg16Arg, Gln27Gln and Gln27Glu genotypes, the risk factor odds ratios were 6.782 (CI = 3.07 to 16.03), 2.120 (CI = 1.22 to 3.71) and 8.096 (CI = 3.90 to 17.77), respectively. For the Gly16Gly and Glu27Glu genotypes, the odds ratios were 0.312 (CI = 0.17 to 0.56) and 0.084 (CI = 0.04 to 0.17), respectively. For more details, consult Tables 1 and 2.

**Table 2:** **Association of *ADRB2 *** **polymorphism [Arg16Gly (c.46A > G) and Gln27Glu (c.79C > G)] combinations with asthma risk**

**Polymorphisms**	Genotypes groups^*^	**Patient**	**Control**	**OR**	**95% CI**
Gly16 – Gln27	1	19	23	1.411	0.71-2.79
Arg16 – Glu27	1	10	0	-	-
Arg16 – Gln27	0	14	5	**5.108**	**1.82-16.37**
Gly16 – Glu27	2	17	11	**2.816**	**1.25-6.54**
Het16 – Gln27	0	10	12	1.376	0.55-3.38
Het16 – Glu27	1	9	0	-	-
Het16 – Het27	0	3	60	**0.048**	**0.01-0.14**
Arg16 – Het27	0	4	4	1.627	0.36-7.39
Gly16 – Het27	1	2	26	**0.1036**	**0.02-0.39**

*ADRB2* = alpha2-adrenergic receptor; Arg = arginine; Gly = Glycine; Gln = Glutamine; Glu = Glutamic acid; A = Adenine; G = Guanine; T = Thymine; Het = heterozygotes; OR = Odds Ratio; CI = Confidence Interval.

*The groups were created by counting each homozygous guanine allele at the Arg16Gly (c.46A > G) and Gln27Glu (c.79C > G) polymorphisms: (0) Arg16Arg + Gln27Gln genotype combination; (1) Gly16Gly or Glu27Glu genotype presence; (2) Gly16Gly + Glu27Glu genotype combination.

The OR was calculated using the Open-Epi tool (http://www.openepi.com). The OR and 95% CI values were obtained using the Mild-P test. For all data analyzed, α = 0.05 was used. The significant p-values are in bold.

The haplotype analysis showed associations between the following polymorphisms: Arg16Arg-Gln27Gln (OR = 5.108, CI = 1.82 to 16.37), Gly16Gly-Glu27Glu (OR = 2.816, CI = 1.25 to 6.54), Arg16Gly-Gln27Glu (OR = 0.048, CI = 0.01 to 0.14) and Gly16Gly-Gln27Glu (OR = 0.1036, CI = 0.02 to 0.39). The complete haplotype analysis is shown in Table 3. To confirm our data, the groups with the highest observed frequency were analyzed in comparison with all of the other possible groups. The complete group data can be found in Table 4.

**Table 3:** **Association of *ADRB2 *** **polymorphisms [Arg16Gly (c.46A > G) and Gln27Glu (c.79C > G)] with asthma risk based on the presence of Guanine alleles**

Genotypes groups*	**Patient**	**Control**	**OR**	**95% CI**
0	31	81	**0.405**	**0.23-0.70**
1	40	49	1.562	0.90-2.70
2	17	11	**2.816**	**1.25-6.54**

*ADRB2* = alpha2-adrenergic receptor; Arg = arginine; Gly = Glycine; Gln = Glutamine; Glu = Glutamic acid; A = Adenine; G = Guanine; T = Thymine; OR = Odds Ratio; CI = Confidence Interval.

*The groups were created by counting each homozygous guanine allele at the Arg16Gly (c.46A > G) and Gln27Glu (c.79C > G) polymorphisms: (0) Arg16Arg + Gln27Gln genotype combination; (1) Gly16Gly or Glu27Glu genotype presence; (2) Gly16Gly + Glu27Glu genotype combination.

The OR was calculated using the Open-Epi tool (http://www.openepi.com). The OR and 95% CI values were obtained using the Mild-P test. For all data analyzed, α = 0.05 was used. The significant p-values are in bold.

**Table 4:** **Association of asthma severity with *ADRB2 *** **polymorphisms [Arg16Gly (c.46A > G) and Gln27Glu (c.79C > G)]**

**Severity**	**Arg16Gly (c.46A > G) polymorphism**							**χ**^**2**^	**p-value**
	**Arg16Arg**	**OR (95% CI)**	**Arg16Gly**	**OR (95% CI)**	**Gly16Gly**	**OR (95% CI)**	**Total**		
**Severe**	14 (37.8%)	1.258 (0.508 - 3.114)	3 (8.1%)	**0.25 (0.064 - 0.966)**	20 (54.1%)	1.672 (0.698 - 4.003)	37 (100%)	4.674	0.322
**Moderate**	7(33.3%)	0.909 (0.319 - 2.587)	5 (23.8%)	1.625 (0.484 - 5.454)	9 (42.9%)	0.703 (0.259 - 1.906)	21 (100%)		
**Mild**	8 (32%)	0.829 (0.306 - 2.246)	7 (28%)	2.431 (0.771 - 7.665)	10 (40%)	0.667 (0.258 - 1.726)	25 (100%)		
	**Gln27Glu (c.79C > G) polymorphism**								
	**Gln27Gln**	**OR (95% CI)**	**Gln27Glu**	**OR (95% CI)**	**Glu27Glu**	**OR (95% CI)**	**Total**		
**Severe**	25 (67.6%)	**2.708 (1.099 - 6.674)**	3 (8.1%)	**0.182 (0.048 - 0.691)**	9 (24.3%)	1.023 (0.372 - 2.812)	37 (100%)	8.285	0.082
**Moderate**	10 (47.6%)	0.701 (0.26 - 1.892)	6 (28.6%)	1.667 (0.534 - 5.197)	5 (23.8%)	0.979 (0.307 - 3.124)	21 (100%)		
**Mild**	10 (40%)	0.438 (0.168 - 1.141)	9 (36%)	**3.063 (1.037 - 9.041)**	6 (24%)	0.9925 (0.331 - 2.973)	25 (100%)		
	Arg16Gly (c.46A > G) and Gln27Glu (c.79C > G) polymorphisms in combination*								
	**0**	**OR (95% CI)**	**1**	**OR (95% CI)**	**2**	**OR (95% CI)**	**Total**		
**Severe**	12 (32.4%)	0.571 (0.232 - 1.406)	21 (56.8%)	1.706 (0.712 - 4.086)	4 (10.8%)	0.994 (0.247 - 4)	37 (100%)	1.908	0.753
**Moderate**	9 (42.9%)	1.188 (0.435 - 3.241)	10 (47.6%)	0.909 (0.338 - 2.448)	2 (9.5%)	0.827 (0.158 - 4.331)	21 (100%)		
**Mild**	12 (48%)	1.626 (0.629 - 4.205)	10 (40%)	0.581 (0.224 - 1.504)	3 (12%)	1.182 (0.271 - 5.154)	25 (100%)		

*ADRB2* = alpha2-adrenergic receptor; Arg = arginine; Gly = Glycine; Gln = Glutamine; Glu = Glutamic acid; A = Adenine; G = Guanine; T = Thymine; OR = Odds Ratio; CI = Confidence Interval.

*The groups were created by counting each homozygous guanine allele at the Arg16Gly (c.46A > G) and Gln27Glu (c.79C > G) polymorphisms: (0) Arg16Arg + Gln27Gln genotype combination; (1) Gly16Gly or Glu27Glu genotype presence; (2) Gly16Gly + Glu27Glu genotype combination.

The OR was calculated using the Open-Epi tool (http://www.openepi.com). The OR and 95% CI values were obtained using the Mild-P test and in the table the χ^2^ value is shown. For all data analyzed, α = 0.05 was used. The significant p-values are in bold.

**Figure 1:**
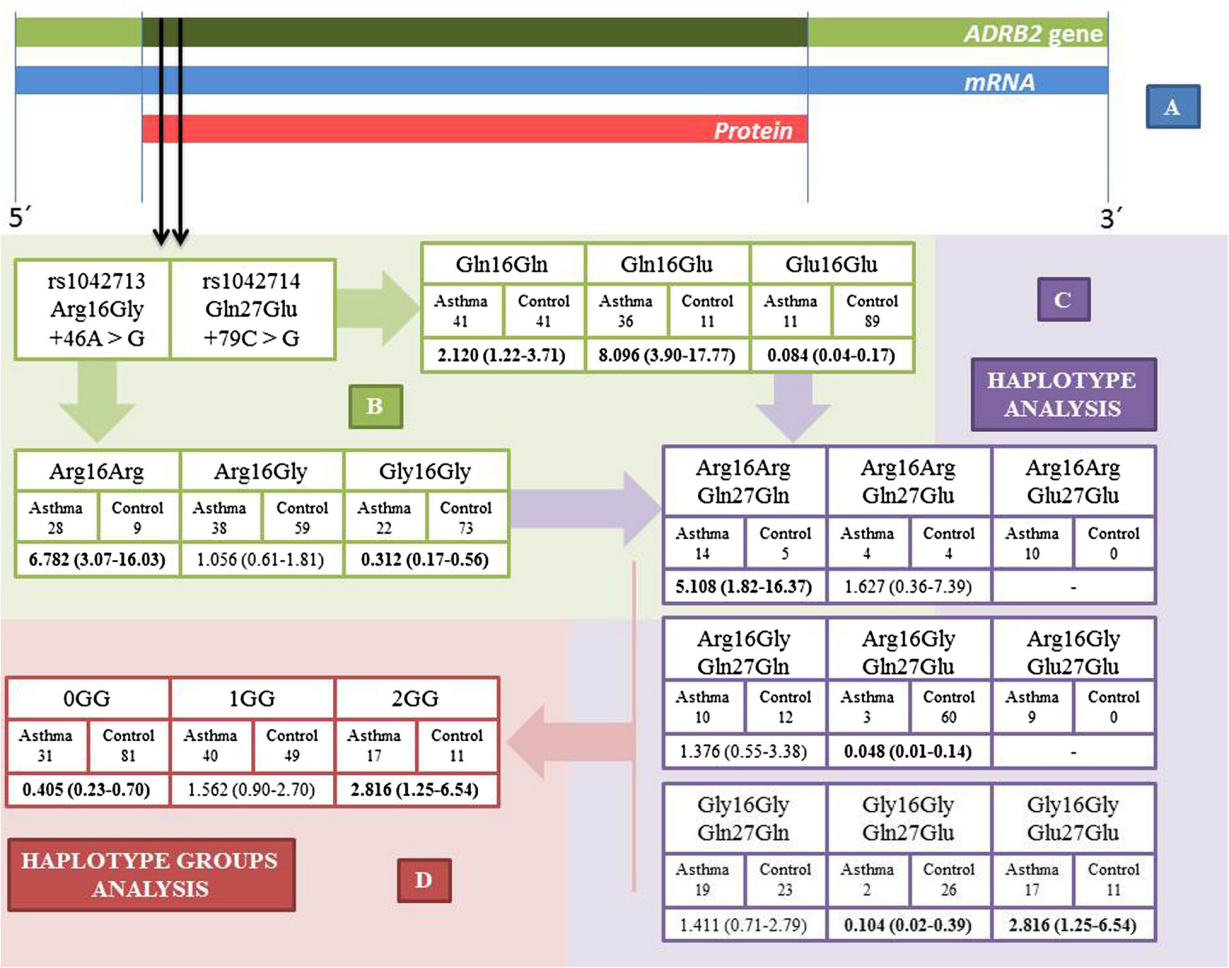
**Complete association of the *ADRB2 *polymorphisms [Arg16Gly (c.46A > G) and Gln27Glu (c.79C > G)] with asthma risk. (A)** Gene, mRNA and protein representation; **(B)** polymorphism analyses (green); **(C)** haplotype analyzed (purple); **(D)** haplotype groups analyzed (red).

All of the data and the comparisons between the groups can be found in Figure 1.

When asthma severity was taken into account, the polymorphism Gln27Glu was a risk factor for severe asthma when the Gln27Gln genotype was present (OR = 2.798, CI = 1.099 to 6.674) and a protective factor for mild (OR = 3.063, CI = 1.037 to 9.041) and severe asthma (OR = 0.182, CI = 0.048 to 0.691) when the Gln27Glu genotype was present.

**Figure 2:**
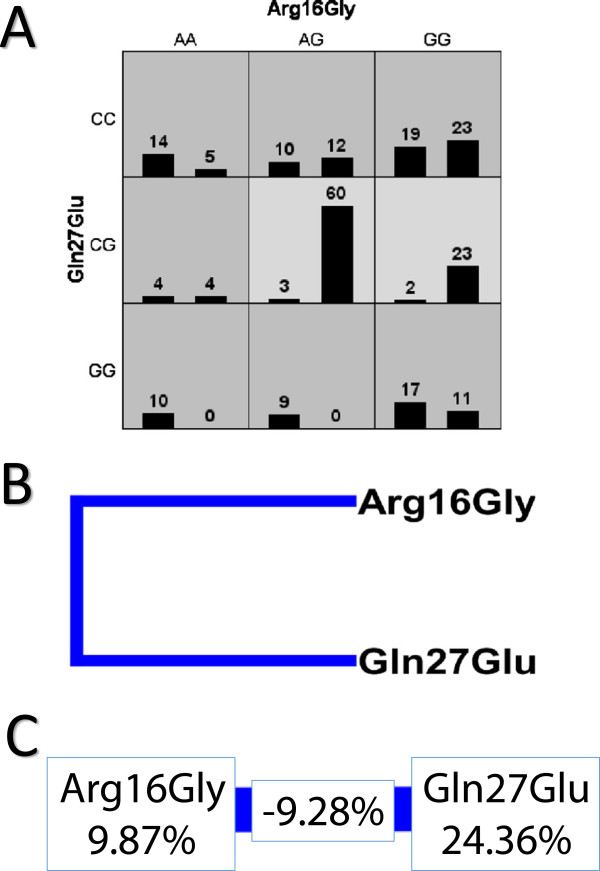
**Multifactor dimensionality reduction test for the Arg16Gly and Gln27Glu polymorphisms in the ADRB2 gene in Asthma patients. A**. Distribution of patients according to different genotype combinations for the clustering of Arg16Gly and Gln27Glu polymorphisms in the *ADRB2* gene. Combinations of high risk are in gray and low risk are in white. The number in the figure represents the patients with a given genotype combination. For example, in the first square, 14 asthma patients (left column) and five healthy patients (right column) have the following genotype: AA for the Arg16Gly polymorphism and CC for the Gln27Glu polymorphism. In this case, the first column in each square represents the asthma patient group, and the second column represents healthy subjects. **B**. Dendrogram of the polymorphism interactions with respect to asthma presence. The same color in this case indicates linkage between the analyzed polymorphisms. **C**. Graph of entropy measuring the power of different polymorphisms and the interactions between them for the gene analyzed to explain the polymorphism-polymorphism association with asthma occurrence. The association is represented by 9.87% for the Arg16Gly polymorphism and 24.36% for the Gln27Glu polymorphism. The interaction between the polymorphisms accounts for -9.28% of the association. The protective genotypes in our samples are CG (for the Gln27Glu polymorphism) and AG or GG (for the Arg16Gly polymorphism).

The MDR analysis showed evidence of an interaction between Arg16Gly and Gln27Glu as risk factors for asthma [Testing Balance Accuracy = 0.7727; p-value = 0.0000 – 0.0010; Ratio = 0.6377] (Figure 2).

## Discussion

Pharmacotherapy that is tailored to an asthmatic patient’s genotype should result in a clinically significant increase in efficacy and reduction in adverse events and, therefore, have an important role in disease severity [37]. The β-agonists are the most commonly used agents for asthma treatment [1]. Polymorphisms in the *ADRB2* gene have been screened and found to be associated with altered expression, function and regulation of the β2 receptor. These types of genetically based differences may account for some of the variability in the responses to treatment with ADRB2 agonists and may contribute to the increased mortality in select patient populations, such as cystic fibrosis patients [31]. Several studies have examined the *ADRB2* gene as a risk factor associated with bronchodilator response [38-40] but not as a risk factor associated with asthma prevalence within a population.

The allelic frequencies of the Arg16Gly and Gln27Glu SNPs vary with ethnicity [41,42]. The reported allele frequencies for Arg16 in the Caucasian, African American and Asian asthmatic populations were 0.39, 0.50 and 0.40, respectively, while for Gln27, the reported frequencies were 0.57, 0.73 and 0.80, respectively [41]. In the present study, the allelic frequencies of Arg16 were 0.53 in the asthma group and 0.27 in healthy subjects. For the Gln27 allele, the allelic frequencies were 0.67 and 0.33 in the asthma group and the healthy subjects, respectively. We observed that the frequencies found in our study are similar to those found in the African American and Caucasian populations.

The Arg16Gly and Gln27Glu polymorphisms cause differential agonist-stimulated down-regulation of the receptor in transfected cell systems, including human airway smooth muscle cells [43,44]. Many previous studies have investigated possible associations between asthma and polymorphisms in the coding region of the *ADRB2* gene, particularly the Arg16Gly and Gln27Glu SNPs; however, these studies have yielded conflicting results [38-40,45-48].

In the present study, associations between the Arg16Arg and Gln27Gln genotypes and susceptibility to asthma were observed.

The Arg16Arg genotype was more frequent in asthma patients than in healthy subjects; the opposite correlation was observed for the homozygous Glu16Glu genotype showing that individuals with the former genotype have an increased susceptibility to the development of asthma. The Gln27Gln and Gln27Glu genotypes were indirectly related to the occurrence of asthma by the fact that the Glu27Glu genotype had a protective effect against asthma. Reinforcing this finding, elevated serum IgE levels have been found in patients carrying the Arg16 and Gln27 homozygous genotypes [49].

Our results contradicted previous data from studies of Japanese [50], African American [51] and North Indian [52] populations but agreed with other studies of Canadian [46], Chinese [53] and British populations [54], as well as a study of African American children [55]. This discrepancy may be the result of racial differences [48].

As expected, the results of the haplotype analysis showed that the haplotype Arg16Arg-Gln27Gln was associated with greater risk and that the Gly16Gly-Glu27Glu haplotype was protective. The haplotype Arg16Arg-Gln27Gln is associated in general with a poor response to the β2-AR agonist and low levels of β2-AR expression. In addition, the good response to exogenous agonists is reflected in a good response to endogenous agonists and a protective effect against asthma [56].

In a case–control study in the North Indian population, the Gly16Gly genotype conferred a decreased risk of asthma (OR = 0.65; 95% IC = 0.41 – 1.02; p-value = 0.049), while the Gln27Glu polymorphism was not associated with asthma in this population [38]. In our study, we observed a positive association between the Arg16Gly polymorphism and asthma prevalence, but the association is weak. These data do not corroborate another study in a Chinese population in which the Arg16Gly polymorphism was not associated with genetic susceptibility to childhood asthma [39]. A contrasting study showed different evidence: increased risk of nocturnal asthma in Egyptian children was associated with the Gly/Gly genotype of the Arg16Gly polymorphism (OR = 3.2; 95% CI = 1.3–7.7; p-value = 0.03) [40]. In this Egyptian study, as in previous studies, the Gln27Glu polymorphism did not show evidence of association with asthma. In this study, the population analyzed should be considered an important environmental factor that interacts with the polymorphisms in the *ADRB2* gene.

Specific data can be reviewed for the polymorphism-associated responses to short- and long-acting β_2_-agonists. For long-acting β_2_-agonists, results have shown no positive association between the Arg16Gly polymorphism and bronchodilation, but the Arg16 allele was associated with poor asthma control [57]. Contrasting results were observed in a Chinese population study. In that study, a significantly higher bronchodilator response was observed in patients with the homozygous genotype 46A/A (13.40% ± 3.48%) compared with those patients with the homozygous genotype 46G/G (7.25% ± 3.11%) and the heterozygous genotype 46A/G (7.39% ± 3.14%) (p < 0.0001) [58]. To determine the effects of the polymorphisms on asthma response to bronchodilators, new studies should be performed that include different populations, higher sample numbers and a complete *ADRB2* gene polymorphism analysis. For the direct response to methacholine, no association was found [59].

Based on the data, no consensus has been reached on the relationship between the identified *ADRB2* genetic variations and asthma. The causal alleles that are common in most ethnic groups may have differential effects because of interactions with the environment and/or other genetic variants that are unique to certain ethnic groups. The interpretation of the findings of the genetic association studies of the *ADRB2* polymorphisms is complicated by the inadequate measurement of environmental exposures and differences in the allele and haplotype frequencies of the *ADRB2* gene and asthma severity among different racial groups. The complexity of the observed genotype-response effects limits their clinical applications [60]. In this context, our study has several strengths and limitations: our sample size may be considered small; there is no control for environmental factors; only two polymorphisms were analyzed; the Brazilian population is admixed; and a region with a specific genotype combination associated with risk may also be associated with a peculiar environmental factor.

The contradictory findings in studies of literature, including the present manuscript, may be associated by: (i) difference in approach to clinical management between centers, (ii) criteria for diagnosis of asthma, (iii) enrolled population of patients (atopic and non-atopic), (iv) population analyzed considering ethnic differences that can alter the genotypic frequency of polymorphisms, (v) clinical variables considered as risk factor (IgE values changed, lung function test, time to diagnosis, evidence of reversibility on spirometry), (vi) presence of non-reported comorbidities, (vii) the characterization of patients taking into account the referral center, whereas non-random sampling for the clinical severity of asthma; (viii) technique for evaluation of polymorphisms in the *ADRB2* gene may have, on rare occasions, erroneous results.

In conclusion, our data show that the Gln27Glu and Arg16Gly polymorphisms of the beta 2-adrenergic receptor gene play an important role in asthma prevalence and severity and are a potential tool for risk analysis in our population. The results reveal the influence of each polymorphism alone and together as a haplotype.

## Abbreviations

ADRB2: Beta-2-adrenergic receptor; cAMP: Cyclic adenosine monophosphate; CI: Confidence interval; OEGE: Online Encyclopedia for Genetic Epidemiology; SPSS: Statistical Package for the Social Sciences; Unicamp: State University of Campinas; β2AR: β2 receptors.

## Competing interests

The authors declare that they have no competing interests.

## Authors’ contributions

CZP, FALM: made substantial contributions to conception and design, acquisition of data, and analysis and interpretation of data; involved in drafting the manuscript and revising it for critically important intellectual content. JDR, CSB: made substantial contributions to conception and design, acquisition of data, and analysis and interpretation of data; involved in drafting the manuscript and revising it for critically important intellectual content. In addition, they have given final approval for the publishing of this version. All authors read and approved the final manuscript.
